# Lysosomal dysfunction of corneal fibroblasts underlies the pathogenesis of Granular Corneal Dystrophy Type 2 and can be rescued by TFEB

**DOI:** 10.1111/jcmm.15646

**Published:** 2020-07-15

**Authors:** Seung‐il Choi, Jong Hwan Woo, Eung Kweon Kim

**Affiliations:** ^1^ Corneal Dystrophy Research Institute Yonsei University College of Medicine Seoul South Korea; ^2^ Department of Ophthalmology Yonsei University College of Medicine Seoul South Korea; ^3^ Institute of Vision Research Yonsei University College of Medicine Seoul South Korea

**Keywords:** autophagy, cathepsin, corneal fibroblasts, granular corneal dystrophy type 2, LC3 degradation, lysosomal pH, TGFBIp

## Abstract

Granular corneal dystrophy type 2 (GCD2) is the most common form of transforming growth factor β‐induced (TGFBI) gene‐linked corneal dystrophy and is pathologically characterized by the corneal deposition of mutant‐TGFBIp. The defective autophagic degradation of pathogenic mutant‐TGFBIp has been shown in GCD2; however, its exact mechanisms are unknown. To address this, we investigated lysosomal functions using corneal fibroblasts. Levels of cathepsins K and L (CTSK and CTSL) were significantly decreased in GCD2 cells, but of cathepsins B and D (CTSB and CTSD) did not change. The maturation of the pro‐enzymes to their active forms (CTSB, CTSK and CTSL) was inhibited in GCD2 cells. CTSL enzymes directly degraded both LC3 (autophagosomes marker) and mutant‐TGFBIp. Exogenous *CTSL* expression dramatically reduced mutant‐TGFBIp in GCD2 cells, but not TGFBIp in WT cells. An increased lysosomal pH and clustered lysosomal perinuclear position were found in GCD2 cells. Transcription factor EB (TFEB) levels were significantly reduced in GCD2 cells, compared to WT. Notably, exogenous *TFEB* expression improved mutant‐TGFBIp clearance and lysosomal abnormalities in GCD2 cells. Taken together, lysosomal dysfunction in the corneal fibroblasts underlies the pathogenesis of GCD2, and TFEB has a therapeutic potential in the treatment of GCD2.

## 1 INTRODUCTION

TGFBI‐linked corneal dystrophy is an autosomal dominant disorder, caused by mutations in the transforming growth factor β‐induced (*TGFBI*) gene on chromosome 5q31, while granular corneal dystrophy type 2 (GCD2) is only caused by an arginine to histidine substitution at codon 124 of the *TGFBI* gene.^1^ GCD2 is pathologically characterized by the age‐dependent progressive accumulation of mutant‐TGFBI proteins (TGFBIp), in the corneal epithelium and stroma, followed by interference in the transparency of the cornea.^1^ Although the worldwide frequency of these disorders is not known, an epidemiological study of the population of South Korea reported an estimated frequency of 11 in 10 000 for GCD2.^2^

The avascular tissue of the cornea is transparent at the frontal surface of the eye and consists of three major layers: the outer epithelium, a thick stroma with keratocytes also known as corneal fibroblasts and the inner endothelium.^3^ Keratocytes are normally quiescent and generally only become activated after corneal injury.^3^ The role of keratocytes in the corneal stroma is to maintain corneal transparency and structures through the degradation and synthesis of cornea‐specific extracellular matrix (ECM) components^4^;damage to these cells may result in impaired vision.

Autophagy is a major intracellular degradation and recycling system that is ubiquitous in eukaryotes. This catabolic process is a response to cellular stress and pathophysiological conditions, whereby cellular organelles and components are engulfed into double‐membrane vesicles called autophagosomes and eventually delivered to lysosomes for degradation.^4,5^ The final step of autophagy is the degradation of the cargo molecules within the lysosomes.

Lysosomes are membrane‐enclosed cellular organelles that consist of two types of lysosomal proteins: lysosomal acid hydrolases and lysosomal membrane proteins. The lysosomal acid hydrolases are involved in the degradation of lysosome cargo and there are more than 60 different types that degrade proteins, nucleic acids, carbohydrates and lipids.^6^ Among them, the cathepsins (CTSs) are a major class of lysosomal protease, which are especially important for autophagy.^7^, ^8^, ^9^ CTSs family consists of aspartic, cysteine and serine CTSs, and they are synthesized as immature (inactive) pro‐CTSs that are proteolytically processed to form mature (active) CTSs.^10^, ^11^ Most lysosomal CTSs are functionally processed to their mature forms (or active form) at acidic pH, as CTSs are stable and active at a low pH.

TGFBIp, which is ubiquitously expressed,^12^ enters through the autophagy pathway^13^ and caveolae‐mediated endocytosis^14^ into lysosomes, where the proteins are degraded. In GCD2 corneal fibroblasts, mutant‐TGFBIp accumulates in the lysosomal compartments due to defective autophagy.^13^, ^15^, ^16^, ^17^ Furthermore, this accumulation of mutant‐TGFBIp leads to cell death of corneal fibroblasts.^13^ Several studies have found that the reduction and clearance of mutant‐TGFBIp in the corneal fibroblasts is a viable therapeutic strategy for the treatment of *TGFBI*‐linked dystrophic patients.^16^, ^17^, ^18^, ^19^ However, there are no therapies or drugs currently available for the treatment of GCD2.

We hypothesized that the accumulation of the pathogenic mutant‐TGFBIp in the corneal fibroblasts may be caused by lysosomal abnormalities and that the enhancement of lysosomal function might counteract the progression of TGFBI‐linked corneal dystrophies. In this study, we tested the lysosomal function of the corneal fibroblasts and the therapeutic effects of exogenous TFEB expression and evaluated the efficacy of this treatment strategy on GCD2 corneal fibroblasts.

## 2 MATERIALS AND METHODS

### 2.1 Antibodies, inhibitors and treatments

All antibodies, reagents and inhibitors that were used in this investigation are listed in Tables S1 and S2. All inhibitors and drugs were dissolved in dimethyl sulfoxide. After 16‐24 hours subcultures, corneal fibroblasts were treated with each inhibitor and the relevant drugs in the fresh growth medium.

### 2.2 Culture of corneal fibroblasts

Primary cultured corneal fibroblasts were prepared from heterozygous (HT) or homozygous (HO) GCD2 patients and normal (wild‐type, WT) healthy corneas from the eye bank, which were obtained during penetrating or lamellar keratoplasty. Donor confidentiality was maintained according to the Declaration of Helsinki and was approved by Severance Hospital IRB Committee (CR04124), Yonsei University. GCD2 was diagnosed by DNA sequencing analysis of *BIGH3* gene mutations. This study used WT (n = 4), HT (n = 1), and HO (n = 3) human corneal fibroblasts, which were immortalized by expression of the catalytic subunit of human telomerase (hTERT).^14^ Corneal fibroblasts were cultured in Dulbecco's modified Eagle's medium (Corning, Manassas, VA, USA) supplemented with 10% FBS (Corning), 100 IU/mL penicillin (Corning), and 100 mg/mL streptomycin (Corning) at 37°C in a humidified incubator with 95% air and 5% CO_2_.

### 2.3 CTSL and TFEB retrovirus plasmid construction and transduction

Each human full‐length TFEB ORF cDNA clone was obtained from OriGene Technologies (SC122773: OriGen Rockville, MD, USA) and the human full‐length CTSL ORF cDNA cloned from the total RNA of the corneal fibroblasts were cloned into the pcDNA3.1 TOPO vector (Invitrogen, Carlsbad, CA, USA) using standard RT‐PCR technology. Supporting information provides more additional detail methods (Methods S1).

### 2.4 Preparation of cell lysates, Western blots and immunoprecipitation analysis

Cell lysates from corneal fibroblasts were prepared in a radio‐immunoprecipitation assay buffer (150 mmol/L NaCl, 1% NP‐40, 0.5% deoxycholate, 0.1% SDS, 50 mmol/L Tris‐HCl, pH 7.4) containing a protease inhibitor (Complete Mini Protease Inhibitor Tablet, Roche #1836170). Supporting information provides more additional detail methods (Methods S2).

For immunoprecipitation, cell lysates were each divided into two equal concentrations and immunoprecipitated with anti‐TFEB or anti‐14‐3‐3 and Dynabeads coated with sheep anti‐mouse IgG (Invitrogen). The immunoprecipitated proteins were analysed by Western blots.

### 2.5 Immunofluorescence staining and confocal microscopy

Corneal fibroblasts were grown on culture slides (BD Falcon Labware, REF 354108) that were permeabilized and fixed in methanol at −20°C for 3 minutes. Supporting Material provides more detail methods (Methods S3).

### 2.6 Acridine orange staining

It has been established that acridine orange accumulates in acidic organelles.^20^ Cells were cultured on a cover glass slide chamber, followed by the designated treatments. Briefly, corneal fibroblasts were exposed to 0.5 μg/mL acridine orange (Sigma‐Aldrich) for 15 minutes at 37°C.^21^ After washing with PBS three times to remove excess acridine orange, the lysosomal structures were visualized with a Zeiss LSM700 confocal microscope (Carl Zeiss).

### 2.7 Determination of the lysosomal pH with a pH indicator

Changes in lysosomal pH were evaluated using the LysoSensor Yellow/Blue DND‐160 reagents following the manufacturer's instructions. Cells (1 × 10^6‐7^/mL) were exposed to Lysosensor yellow/blue DND‐160 (Invitrogen) at a final concentration of 10 μmol/L for 1 hour in PBS. Cells were then washed three times with ice‐cold PBS and kept on ice until just before starting observation.

### 2.8 RNA isolation and quantitative real‐time PCR

Total RNA was isolated as described by the manufacturer using Trizol reagent (Invitrogen). Quantitative real‐time PCR (RT‐qPCR) was performed using Taq‐Man^®^ Universal PCR Master Mix II (Applied Biosystems) with specific primers (Table S3). The mRNA levels β‐actin and GAPDH were used to normalize the expression of target genes. Relative quantification was performed using system software based on the 2^−DDCt^ method.

### 2.9 Subcellular fractions

Nuclear fractions and the cytoplasm were prepared using a NE‐PER® nuclear and cytoplasmic extraction reagents (Pierce, 78833) according to the manufacturer's protocol. Supporting information provides more detail methods (Methods S4).

### 2.10 In vitro cleavage assays of TGFBIp and LC3

Human CTSL (C6854: Sigma‐Aldrich) was used to digest TGFBIp and LC3 proteins in vitro. Supporting information provides more detail methods of in vitro cleavage assay (Methods S5).

### 2.11 Imaging

For analysis of the TFEB in the nucleus, Z‐stack images were captured at ×20 and ×60 magnifications using the Zeiss LSM 700 confocal microscope (Carl Zeiss) and analysed using the Zeiss LSM ZEN 2012 software (Carl Zeiss).

### 2.12 Statistics

Statistical significance was assessed by two‐tailed Student's *t* test or one‐way ANOVA (for multiple comparisons) using the scientific graphing analysis software (Prism, version 5.0; GraphPad Software Inc, San Diego, CA, USA). *P* values < 0.05 were considered statistically significant.

## 3 RESULTS

### 3.1 Altered processing and levels of CTSs in GCD2 corneal fibroblasts

**FIGURE 1:**
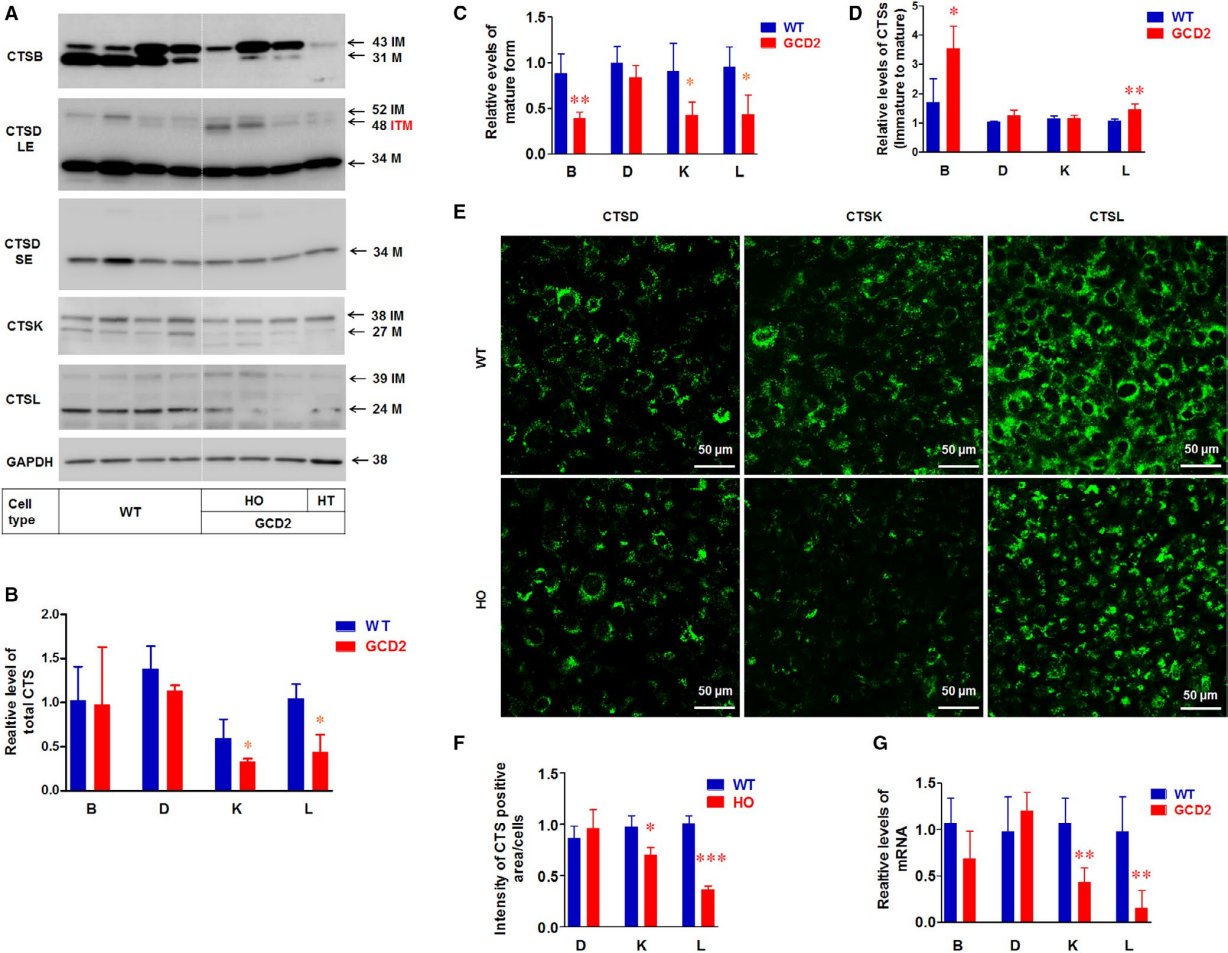
Altered levels and maturation of cathepsins (CTSs) in GCD2 corneal fibroblasts. A, Western blotting analysis of CTSB, CTSD, CTSK, CTSL and GAPDH in WT and GCD2 corneal fibroblasts. WT, wild‐type corneal fibroblasts; HO, homozygous corneal fibroblasts; HT, heterozygous corneal fibroblasts; GCD2, granular corneal dystrophy type 2; LE, long‐short exposure; SE, short exposure; IM, immature form (or inactive form); ITM, intermediate form (intermediate proenzyme); M, mature form (or active form); kD, kilodalton. B, Quantification of relative band intensities including IM, ITM, and M forms (total levels) of CTSs in (A). Data were normalized against GAPDH (n ≥ 3). C, Quantification of relative band intensities of matured forms (M) of CTSs in (A). Data were normalized against GAPDH (n ≥ 3). D, Quantitative ratio of relative band intensities of immature (IM) of CTSs in (A). Data were normalized against GAPDH (n ≥ 3). E, Representative immunofluorescence images of STSD, CTSK, and CTSL in WT and HO corneal fibroblasts. F, Quantification of staining intensities of CTSD, STSK and CTSL in (E). At least 30 corneal fibroblasts were analysed for each group from three independent experiments. G, Transcripts levels of CTSB, CTSD, CTSK and CTSL. The mRNA expression of CTSs in WT and GCD2 corneal fibroblasts was analysed by real‐time RT‐PCR. Data were normalized against β‐actin (n = 3). Statistical significance was determined by Student's t test. Data represent the mean ± SD. *P ≤ 0.05; **P ≤ 0.01; ***P ≤ 0.001. NS, not significant

Our previous study showed that the pathogenic mutant‐TGFBIp accumulates in the lysosomal compartments of GCD2 corneal fibroblasts.^13^ This study indicates that lysosomes may be functionally abnormal in GCD2 corneal fibroblasts. Accordingly, we first analysed the levels of CTSs. The results showed that the levels of CTSK and CTSL were significantly reduced in GCD2 corneal fibroblasts compared to WT, but the levels of CTSB and CTSD did not differ between the two cells (Figure 1A,B). We also measured the levels of CTSs that were in their active form. Western blot analysis showed that the active or matured forms (M) of CTSB, CTSK and CTSL were significantly decreased in GCD2 corneal fibroblasts, compared with WT (Figure 1A,C). In contrast, the levels of CTSD did not change (Figure 1A,B). We also analysed the ratio of the immature (IM) CTS to the active form (Figure 1A). The ratios of immature CTSB and CTSL to their active forms were significantly increased in GCD2 corneal fibroblasts compared with WT (Figure 1A,D). However, the ratio of the immature forms of CTSD and CTSK to their active forms did not differ between GCD2 and WT corneal fibroblasts (Figure 1A,D). Additionally, an increased level of CTSD intermediate proenzyme (ITM; 48 kDa) was observed in GCD2 HO corneal fibroblasts (Figure 1A).

Confocal microscopy showed much weaker intracellular fluorescence intensity for CTSK and CTSL in GCD2 HO corneal fibroblasts, but not for CTSD (Figure 1E,F). In addition, the mRNA expression analysis from RT‐qPCR showed that the mRNA levels of CTSK and CTSL were significantly reduced, but those of CTSB and CTSD were not significantly different in GCD2 corneal fibroblasts compared with WT (Figure 1G). Because we developed only one line of HT corneal fibroblasts, we limited our comparison between normal and pathological states to WT and HO cells, respectively. Accordingly, all data presented subsequent to Figure 1 are restricted to these cell lines.

### 3.2 Perturbation of lysosomal acidification in GCD2 corneal fibroblasts

**FIGURE 2:**
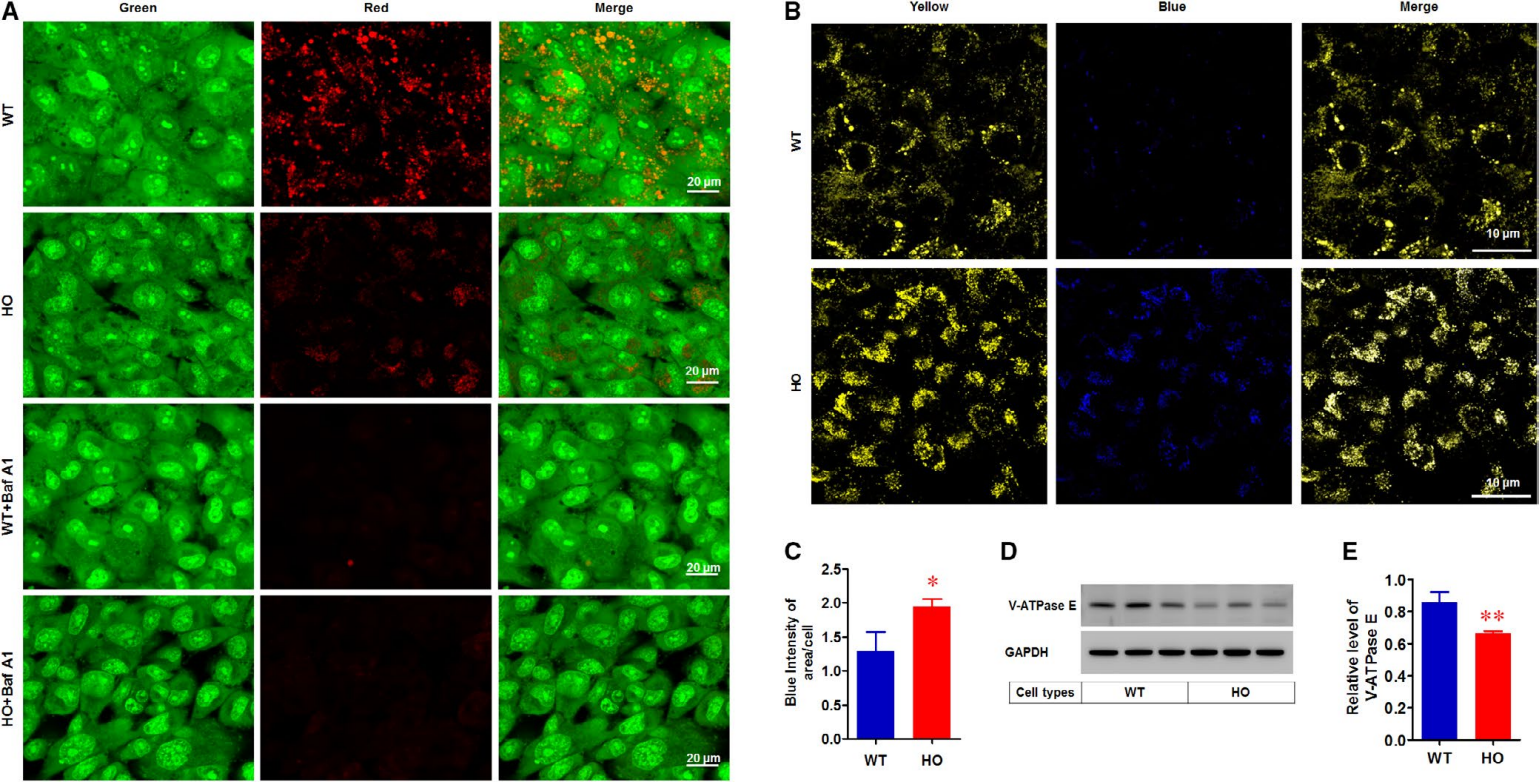
Analysis of lysosomal acidification in GCD2 corneal fibroblasts. A, Acridine orange (AO) uptake in corneal fibroblasts. Representative images of AO‐stained fibroblasts that were untreated or treated with 200 nmol/L Baf A_1_ (Bafilomycin A_1_) for 4 h. Red staining is associated with acidic vesicles, whereas green staining is associated with high pH. Nucleic acids are stained green. Scale bar 20 µm. B, Altered lysosomal pH in WT and GCD2 HO corneal fibroblasts. The corneal fibroblasts were loaded with 5 µmol/L LysoSensor Yellow/Blue DND‐160 for 5 min and immediately imaged under a confocal microscope. Dual‐emission ratio‐metric measurement of lysosomal pH using LysoSensor Yellow/Blue DND‐160. These pseudo‐coloured images were constructed from two emission images. In these images, the fluorescence near pH 4 was pseudo‐coloured yellow‐green and fluorescence near pH 7 was pseudo‐coloured blue. C, Quantification of staining intensities of the LysoSensor Yellow/Blue DND‐160. At least 60 corneal fibroblasts were analysed for each group. D, Western blot analysis of ATPase E levels in WT and GCD2 HO corneal fibroblasts. The total protein extracted from WT and GCD2 HO corneal fibroblasts was analysed by using anti‐V‐ATPase and GAPDH antibodies. GAPDH was used as a loading control. E, Quantification of relative band intensities of V‐ATPase in (D). The bands were normalized against GAPDH (n = 3). Data are representative of three independent experiments. Values indicate the means ± SD. Statistical significance was determined by Student's t test. *P ≤ 0.05; **P ≤ 0.01. NS, not significant

Pro‐CTSs or immature CTSs are proteolytically processed to active or mature CTS forms upon acidification in lysosomes and endolysosomes.^22^, ^23^ Accordingly, high levels of immature CTSs in GCD2 corneal fibroblasts (Figure 1A,D) indicate altered lysosomal acidification. To confirm this, the lysosomal acidification was assessed by vital staining with acridine orange. This dye is an acidotropic weak base, which is taken up by living cells and accumulates in acidified compartments such as the lysosomes.^24^, ^25^ Acridine orange has a green fluorescence at low concentrations and red at high concentrations. Consequently, when corneal fibroblasts were stained with acridine orange, the nuclei and the cytoplasm showed green fluorescence, whereas the acidified lysosomes showed red fluorescence in a granular pattern in the cytoplasm. WT corneal fibroblasts showed intact lysosomal compartments, as indicated by the cytoplasmic red fluorescence (Figure 2A). In contrast, most GCD2 corneal fibroblasts showed markedly decreased red fluorescence (Figure 2A), indicating an elevated lysosomal pH. To further confirm whether the decreased red fluorescence resulted from a loss of lysosomal acidification in GCD2 corneal fibroblasts, we employed bafilomycin A_1_, a potent inhibitor of vacuolar‐type H^+^‐ATPase (V‐ATPase) that indirectly inhibits fusion between the autophagosome and lysosomes by increasing the lysosome pH. As expected, the treatment of both corneal fibroblasts with bafilomycin A_1_ for 1 hour before acridine orange staining caused complete disappearance of the red fluorescence, whereas the green fluorescence remained (Figure 2A). In addition, to examine specifically the luminal pH of the lysosomes, we performed live imaging assays using a pH‐sensitive fluorescent dye, LysoSensor Yellow/Blue DND‐160. This dye is characterized by its spectral properties which produce yellow fluorescence in an acidic environment and blue in an alkaline environment. Confocal imaging of WT and GCD2 corneal fibroblasts treated with the DND‐160 confirmed that most of WT corneal fibroblasts displayed a yellow fluorescence (pH of 4‐5), and GCD2 corneal fibroblasts displayed a blue fluorescence (pH 6‐7) (Figure 2B,C). To further explore the mechanisms underlying the lysosomal acidification in GCD2 corneal fibroblasts, we assayed the level of V‐ATPase, as the lysosomal pH is regulated by components of the V‐ATPase complex that participates in lysosomal acidification.^26^, ^27^ Interestingly, the Western blots showed that the relative level of the E subunit of the V‐ATPase was significantly reduced in GCD2 corneal fibroblasts compared with WT (Figure 2D,E).

### 3.3 Lysosomes are highly concentrated in the perinuclear regions of the GCD2 corneal fibroblasts

**FIGURE 3:**
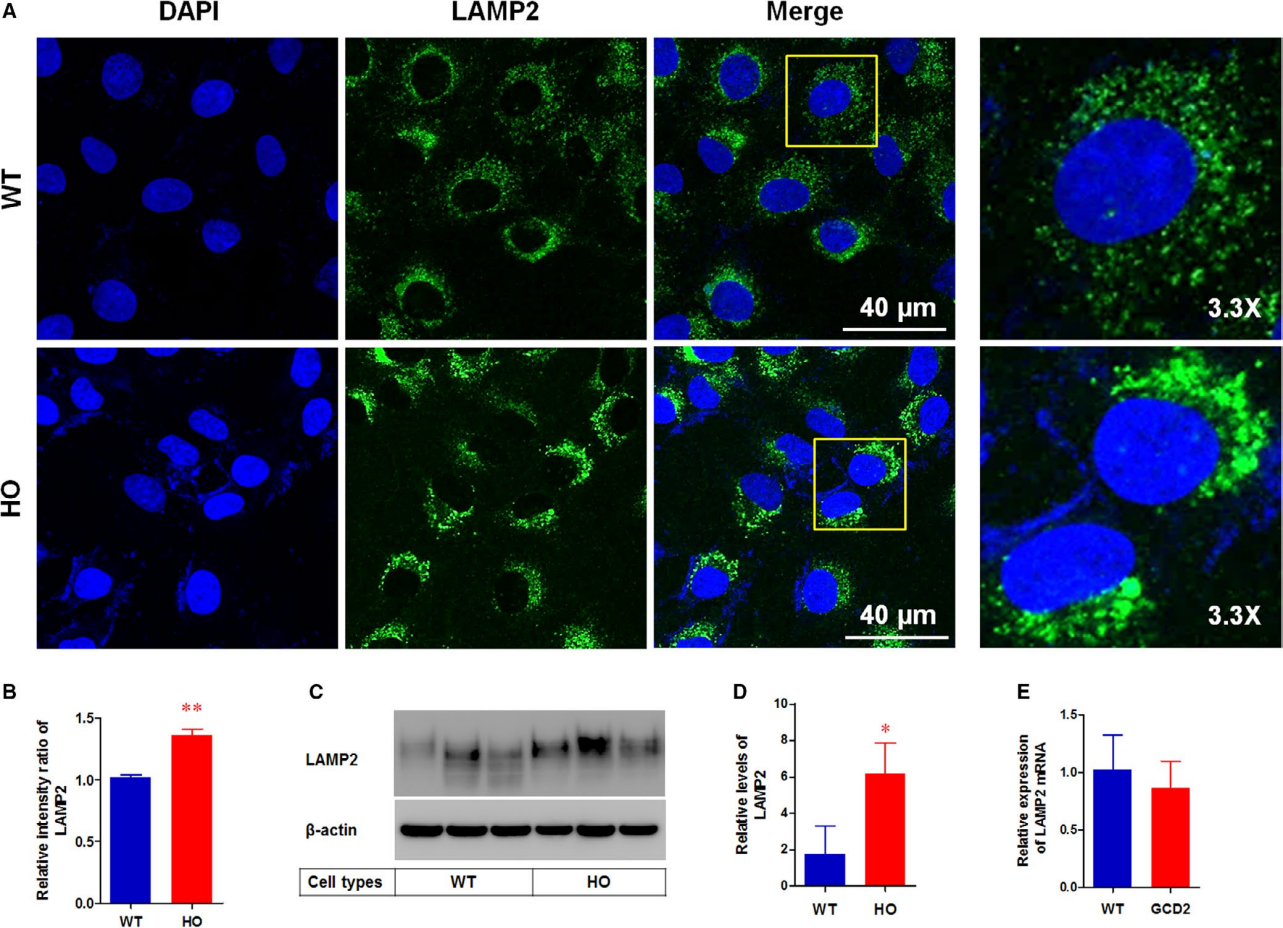
Altered lysosomal distributions in GCD2 corneal fibroblasts. A, Representative images of LAMP2 (lysosomal marker) staining in WT and GCD2 corneal fibroblasts. The corneal fibroblasts were methanol‐fixed and immunostained with anti‐LAMP2 (green: lysosomal marker) and counterstained with DAPI (blue: nuclear marker). B, Quantification of staining intensities of LAMP2 in (A). At least 40 corneal fibroblasts were analysed for each group (n ≥ 3). C, Western blots analysis of LAMP2 levels in WT and GCD2 corneal fibroblasts. D, Quantification of relative band intensities of LAMP2 levels in (C). Data were normalized against β‐actin (n = 3). E, Real‐time RT‐PCR analysis of LAMP2 mRNA levels in WT and GCD2 corneal fibroblasts. Quantification of relative levels of LAMP2 mRNA. Data were normalized against β‐actin (n = 3). Data are representative of three independent experiments for A and C and two independent experiments for E. Differences between the values were analysed by Student's t test. *P ≤ 0.05; **P ≤ 0.01. NS, not significant

The shape and distribution of lysosomes correlated with the changes in the intracellular pH.^28^, ^29^ Therefore, we investigated the distribution of lysosomes. The confocal images showed that the lysosome‐associated membrane protein 2 (LAMP2), a specific lysosomal marker, was distributed in the peripheral area of the WT corneal fibroblasts (Figure 3A). Lysosomal distribution pattern in GCD2 HO corneal fibroblasts was highly concentrated in the perinuclear regions (Figure 3A). Furthermore, quantification of the intensities of LAMP2 staining was significantly elevated in the HO corneal fibroblasts compared to WT (Figure 3B). Western blot analysis also showed that LAMP2 protein levels were significantly increased (Figure 3C,D), whereas mRNA levels in GCD2 corneal fibroblasts did not differ compared with in WT (Figure 3E).

### 3.4 CTSL is the degrading enzyme of TGFBIp and exogenous CTSL expression promotes the clearance of mutant‐TGFBIp

**FIGURE 4:**
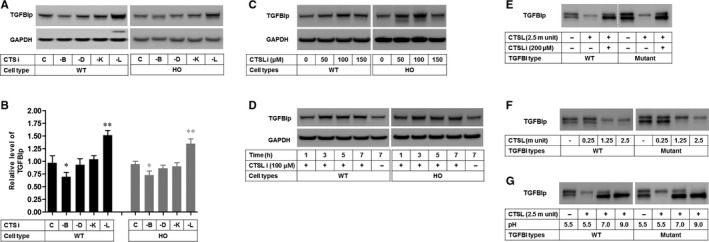
Identification of CTSs in the lysosomal degradation of TGFBIp. A, TGFBIp levels in WT and HO corneal fibroblasts, which were treated with inhibitors of CTSB, CTSD, CTSK and CTSL were analysed by Western blotting with an anti‐TGFBIp antibody. CTS i: CTS inhibitor. B, Quantification of relative band intensities of TGFBIp in (A). Data were normalized against GAPDH (n = 3). (C,D) Western blot analyses were performed to evaluate the dose‐ and time‐dependent effects of CTSL i on TGFBIp levels in WT and GCD2 HO corneal fibroblasts. E, CTSL cleaved TGFBIp and mutant‐TGFBIp. TGFBIp was digested with 2.5 m units of CTSL for 2 h at 37°C in pH 5.5, with or without CTSL i in vitro. An uncleaved TGFBIp control sample was incubated in buffer without enzyme for 2 h at 37°C at pH 5.5. Samples were analysed by Western blotting with an anti‐TGFBIp antibody. −: untreated; +: treated; CTSL i: CTSL inhibitor. (F,G). Western blot analysis showing the effects of different CTSL i doses (F) and pH (G) on the degradation of TGFBIp. TGFBIp and mutant‐TGFBIp were incubated with different concentrations of CTSL and pH for 2 h at 37°C in vitro and analysed by Western blotting. Data are representative of three independent experiments for (A) and two independent experiments for (C–G). Differences between the values were analysed by one‐way ANOVA. *P ≤ 0.05; **P ≤ 0.01. NS, not significant

The delayed degradation of the mutant‐TGFBIp within the lysosomes and the autophagolysosome (Data S1)^13^, ^14^ and the reduced expression of CTSL and CTSK (Figure 1) indicate that CTSL and CTSK could degrade the mutant‐TGFBIp in lysosomes. Accordingly, to investigate whether CTSK and CTSL had the ability to degrade the TGFBIp, we treated several CTS inhibitors with WT and HO corneal fibroblasts, and then assayed the levels of TGFBIp. The results showed that CTSB inhibitor significantly reduced the levels of TGFBIp in both WT and HO corneal fibroblasts (Figure 4A,B). Conversely, CTSL inhibitor resulted in the significant accumulation of TGFBIp in both WT and GCD2 corneal fibroblasts (Figure 4A,B). Additionally, the TGFBIp is accumulated in the presence of CTSL inhibitor, in a dose‐ (50‐150 µmol/L: Figure 4C) and time‐ (1‐7 hours: Figure 4D) dependent manner, in both WT and GCD2 corneal fibroblasts. These results indicate that CTSL may degrade both WT‐ and mutant‐TGFBIp within the lysosomes. Therefore, we investigated whether CTSL could degrade the TGFBIp directly through in vitro cleavage assay. As expected, the treatment with CTSL enzyme resulted in the disappearance of the bands for both the TGFBIp and mutant‐TGFBIp (Figure 4E, lane 2 and 5). Additionally, the smaller unit (0.25) of CTSL did not degrade TGFBIp or the mutant‐TGFBIp (Figure 4F, lane 2 and 6), whereas the medium and large units (1.25 and 2.5) degraded both (Figure 4F, lane 3 and 4, and 7 and 8). Similarly, the degradation of TGFBIp by CTSL at pH 5.5‐9 was determined by Western blotting. It was found that in the acidic lysosome (pH 5.5), CTSL was able to degrade the TGFBIp (Figure 4G, lane 2 and 6) and that there was partial cleavage of the TGFBIp at pH 7‐9 (Figure 4G, lane 3 and 4 in TGFBIp, and lane 7 and 8 in mutant‐TGFBIp). Further, we determined that the optimum pH for CTSL to degrade TGFBIp was 4.5 (Data S2). In addition, we are showed that CTSL is a major degrading enzyme of LC3‐II in autophagolysosome and lysosomes through inhibitor (Data S3A‐F) and in vitro assay with CTSL enzyme (Data S3G‐I).

### 3.5 Exogenous CTSL expression promotes the clearance of pathogenic mutant‐TGFBIp

**FIGURE 5:**
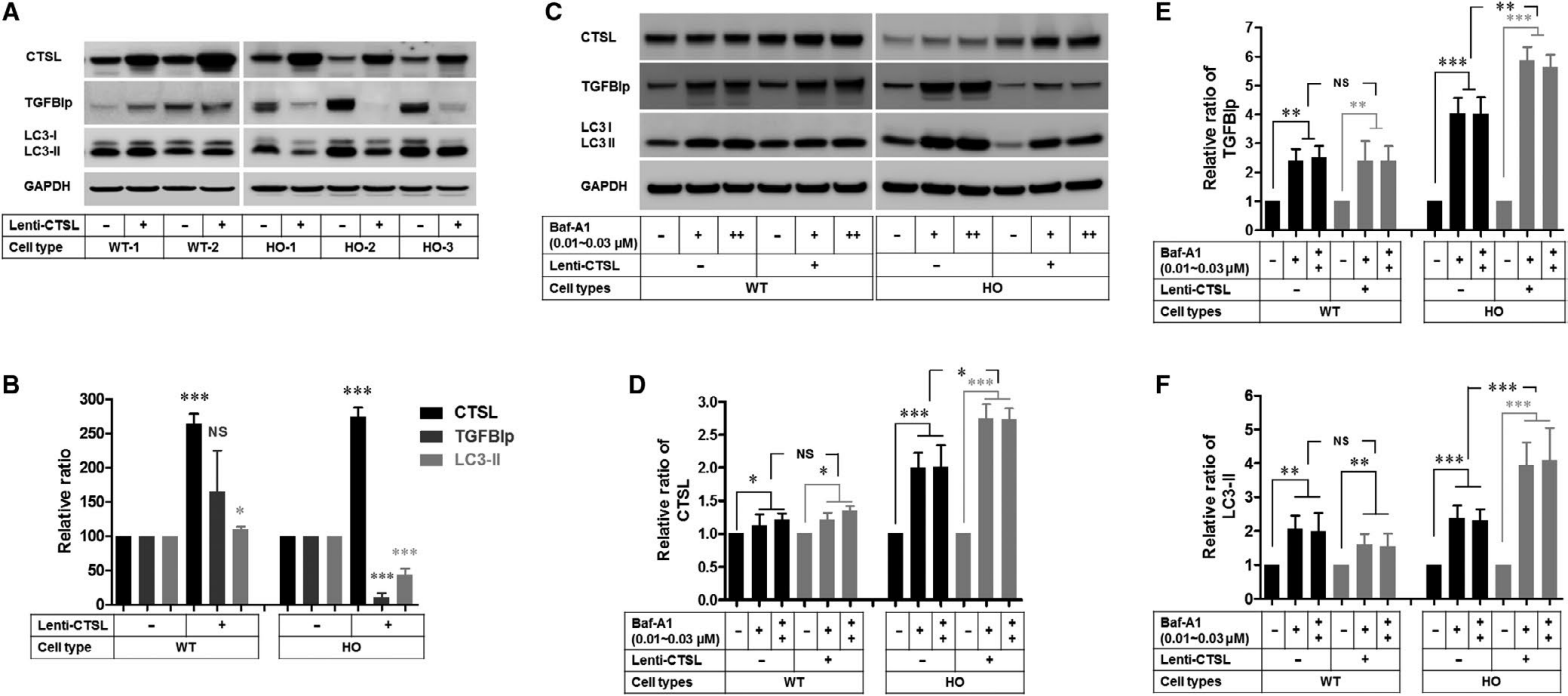
Death of GCD2 corneal fibroblasts are induced by inhibition of CTSB and CTSL. A, Cell viability analysis in WT and GCD2 corneal fibroblasts treated with inhibitors of CTSL and CTSB. WT, HT and HO corneal fibroblasts were exposed to CTSs i for 16 h, and quantitative cell viabilities were estimated using PrestoBlue cell viability reagent (n ≥ 3). B, Apoptosis analysis in WT and GCD2 HO corneal fibroblasts treated with inhibitors of CTSL and CTSB for 16 h. PARP1 and β‐actin were determined by Western blotting. Cleaved PARP1 showed two major fragments migrating at 113 and 89 kD. C, Quantification of relative band intensities of Cl‐PARP1 in B (n = 3). D, Representative phase‐contrast photomicrographs of CTSL i‐treated WT and HO corneal fibroblasts (n ≥ 3). X200. Cl‐PARP1, Cleaved PARP1. CNT, corneal. NS, not significant. Results are representative of three independent experiments. Values are expressed as the mean ± SD. Statistical tests were as follows: one‐way ANOVA for (A) and Student's t test for (C). *P ≤ 0.05, **P ≤ 0.01, ***P ≤ 0.001. NS, not significant

The ability of CTSL to clear mutant‐TGFBIp was then investigated. In this test, to overcome the very low or lack of transfection efficiency of the corneal fibroblasts, we used a lentivirus‐based gene delivery system. First, the lent‐CTSL infection led to the enhancement of the CTSL in both the WT and HO corneal fibroblasts (Figure 5A,B, lanes 2, 4, 6, 8 and 10). Second, we assayed the effects of the CTSL overexpression on the levels of the TGFBIp. Interestingly, the results showed that the mutant‐TGFBIp was significantly reduced in the HO‐Lenti‐CTSL cells (Figure 5A, lanes 6, 8 and 10), but not in the WT‐Lenti‐CTSL corneal fibroblasts (Figure 5A, lanes 2 and 4). Furthermore, enhanced levels of the CTSL led to an increased ratio of LC3‐II/LC3‐I in the WT corneal fibroblasts (Figure 5A, lanes 2 and 4, and Figure 5B, lanes 6), but reduced ratio of LC3‐II/LC3‐I in the GCD2‐Lenti‐CTSL corneal fibroblasts (Figure 5A,B: lanes 6 and 8, and 10). We also investigated whether the reduced mutant‐TGFBIp levels resulted from the induction of autophagy or the activation of CTSL. We used bafilomycin A_1_ in the WT and GCD2 corneal fibroblasts infected with or without Lenti‐CTSL overexpression. Bafilomycin A_1_ significantly enhanced the levels of CTSL, TGFBIp, and LC3‐II in the Lenti‐CTSL infected and none‐infected WT cells, and the GCD2 cells (Figure 5C,D,E,F: lane 2, 3, 5 and 6, and 8, 9, 11 and 12, respectively). Furthermore, bafilomycin A_1_ significantly enhanced the levels of CTSL, TGFBIp and LC3‐II in the Lenti‐CTSL‐infected GCD2 cells, compared with the GCD2 cells, but not the Lenti‐CTSL‐infected WT and WT cells (Figure 5C,D,E,F: lane 1, 2 and 3 compared to 4, 5 and 6, respectively). These data indicate that enhancing CTSL could remove the mutant‐TGFBIp through the activation of autophagy in the GCD2 cells.

Previously, it has been described that apoptosis was induced in the cells expressing antisense CTSL RNA^30^ and in the *CTSL* gene knock‐out mice.^31^ Accordingly, we have examined the effects of CTSs inhibition between the GCD2 and WT cells. These results showed that the cell viability of the GCD2 cells was the most susceptible to CTSL inhibition. Collectively, these results suggest that reduced CTSL with GCD2 may provide the additional cytotoxicity to pathogenic mutant‐TGFBIp (Data S4).

### 3.6 TFEB was reduced in GCD2, and exogenous TFEB expression rescues the lysosomal abnormalities and GCD2 corneal fibroblasts from CTSL inhibitor‐induced apoptosis

**FIGURE 6:**
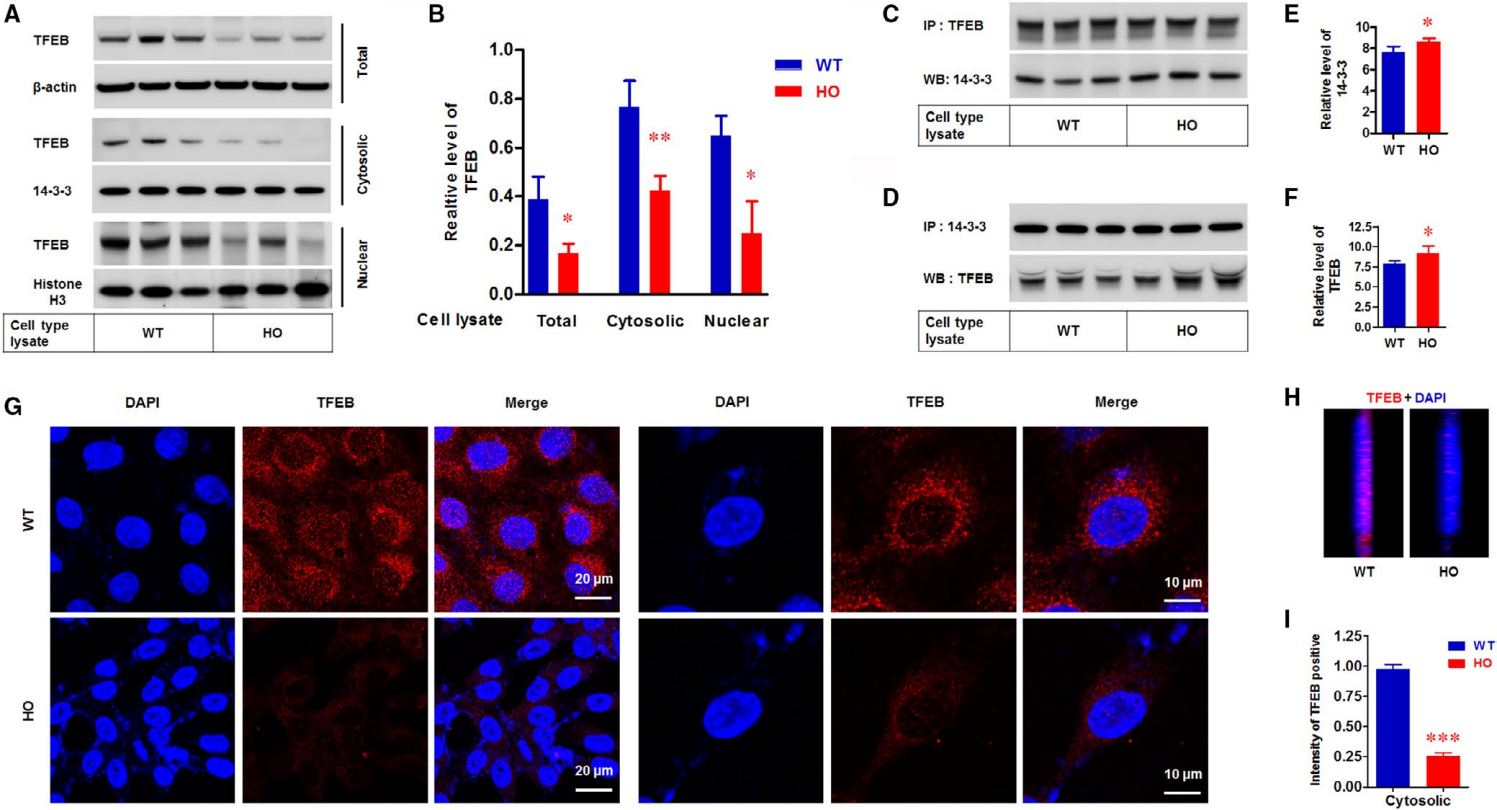
Level and activation of TFEB were reduced in GCD2 corneal fibroblasts. A, Western blot analysis of TFEB protein levels in WT and GCD2 HO in the total cell lysate, cytosolic and nuclear fractions. β‐Actin as loading controls of the total protein, 14‐3‐3 proteins as loading controls of the cytosolic protein, and histone H3 as loading controls of the nucleus protein were detected. WT, WT cell lysate; HO, HO cell lysate. B, Quantification of relative band intensities of TFEB in (A) (n = 3). Quantification of TFEB band intensities was normalized for β‐actin, 14‐3‐3 and histone H3 bands, respectively. WT, WT cell lysate; HO, HO cell lysate. (C,D) Western blotting analysis of co‐immunoprecipitated TFEB with 14‐3‐3 proteins. The extracts of the total protein were co‐immunoprecipitated with anti‐TFEB and anti‐14‐3‐3 protein antibodies, respectively. The immunoprecipitates were analysed by Western blotting with antibodies against 14‐3‐3 proteins (C) and TFEB (D). IP, immunoprecipitation. WB, Western blots. WT, WT cell lysate; HO, HO cell lysate. E, Quantification of band intensities of 14‐3‐3 proteins. Quantification of band intensities was normalized to TFEB (n = 3). WT, WT cell lysate; HO, HO cell lysate. F, Graph showing the quantification of TFEB (D) (n = 3). Quantification of band intensities was normalized to 14‐3‐3 protein. WT, WT cell lysate; HO, HO cell lysate. G, Immunofluorescence confocal microscopy of the subcellular distribution of TFEB in WT and HO corneal fibroblasts. Corneal fibroblasts were fixed, permeabilized with cold methanol and stained with antibodies against TFEB. Bars, 20 µm. IP, immunoprecipitation. H, Immunofluorescence confocal microscopy showed the nuclear distribution of TFEB in WT and HO corneal fibroblasts. I, Quantification of the relative dot number in the nucleus of WT and HO corneal fibroblasts. At least 20 corneal fibroblasts were analysed for each group. Data are representative of three independent experiments. Values are expressed as the mean ± SD. Differences between values were analysed by Student's t test. *P ≤ 0.05; **P ≤ 0.01; ***P ≤ 0.001. NS, not significant

Recently, the TFEB has been identified as a master regulator of lysosomal biogenesis.^26^, ^32^ TFEB regulates the expression of various autophagy‐ and lysosome‐related proteins, including CTSs and ATG.^26^, ^32^ Accordingly, we assayed the levels and activity of TFEB. The result showed that the level of TFBE (Figure 6A,B), the interaction between 14‐3‐3 and TFEB (Figure 6C‐F), and the nuclear localization of TFEB (Figure 6G‐I) was significantly reduced in GCD2 corneal fibroblasts compared with WT cells.

**FIGURE 7:**
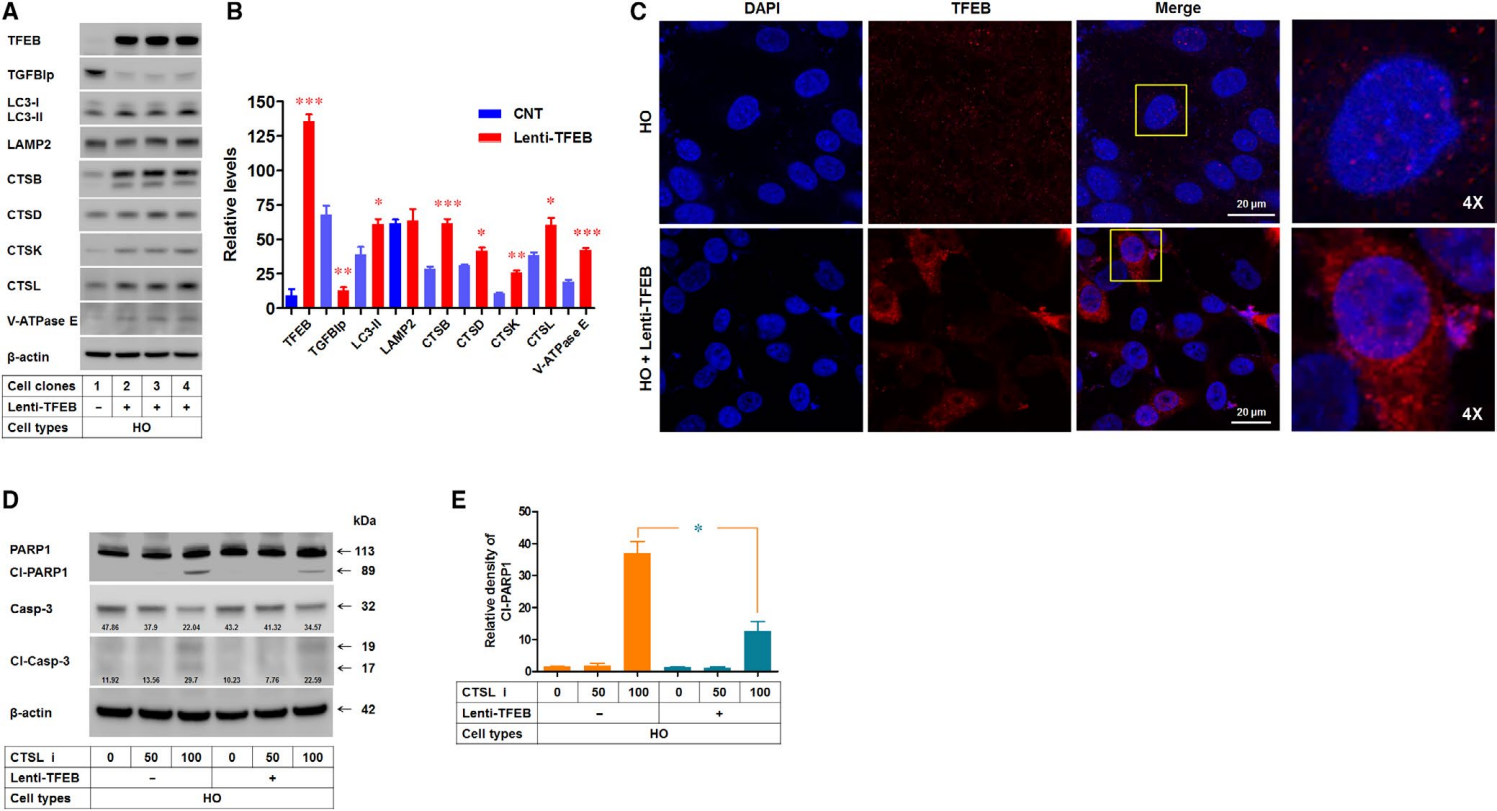
Effects of TFEB expression on mutant‐TGFBIp levels and lysosomal dysfunctions in GCD2 corneal fibroblasts. A, Western blot analysis of TFEB, TGFBIp, LC3, LAMP2, CTSB, CTSD, CTSK, CTSL and V‐ATPase E in HO and HO‐Lenti‐TFEB corneal fibroblasts. B, Quantification of relative band intensities of Western blot results in (A). Quantification of band intensities was normalized to β‐actin band (n = 3). C, Immunofluorescence staining for TFEB in HO and HO‐Lenti‐TFEB corneal fibroblasts. D, Western blots of PARP1, Cl‐PARP1, Casp‐3, Cl‐Casp‐3 and β‐actin in HO and HO‐Lenti‐TFEB corneal fibroblasts. E, Quantification of relative band intensities of Cl‐PARP1 in (D) (n = 3). Data are representative of three independent experiments. Values are expressed as the mean ± SD. Differences between values were analysed by Student's t test. *P ≤ 0.05; **P ≤ 0.01; ***P ≤ 0.001. NS, not significant

TFEB plays an essential role in cellular homeostasis and has provided us with a novel tool to modulate lysosomal biogenesis and function, and autophagy.^24^, ^25^ Accordingly, we examined whether enhancing TFEB could recover the altered CTSs levels and lysosomal dysfunction and if it could also clear mutant‐TGFBIp. Importantly, the exogenous TFEB expression significantly promoted the reduction of mutant‐TGFBIp levels (Figure 7A,B). Western blots also showed that exogenous TFEB expression significantly increased the protein levels of LC3‐II, CTSB, CTSD, CTSK, CTSL and V‐ATPase E, but not CTSB and CTSD (Figure 7A,B). Further, the immunofluorescence confocal images showed greatly enhanced levels of TFEB in the cytosol and nucleus of GCD2 HO‐Lenti‐TFEB corneal fibroblasts compared with in GCD2 HO cells (Figure 7C). Furthermore, TFEB expression significantly inhibited the cleavage of PARP1 and procaspase‐3 induced by CTSL inhibitor (Figure 7D,E).

## 4 DISCUSSION

We here evaluated the lysosomal roles in the intracellular accumulation of mutant‐TGFBIp and concluded that the accumulation of this pathogenic mutant‐TGFBIp by defective autophagy is caused by lysosomal dysfunction and that TFEB activation could be extremely important as a potential therapeutic target in treatments for GCD2.

To study the pathogenesis of GCD2, we established WT, HT and HO lines from primary corneal fibroblasts. Although we found differences between HT and WT primary corneal fibroblasts in previous study,^5^, ^33^ we were unable to determine statistical significance in this study, as only one line of HT cells was established. Consequently, we only used data from WT and HO cells in this study to examine the differences between normal and pathological states.

Our findings demonstrate that altered maturation and reduced levels of CTSs in GCD2 corneal fibroblasts. Considering that CTSL is a TGFBIp‐degrading enzyme, the accumulation of mutant‐TGFBIp in lysosomes and autophagolysosomes^13^ could be caused by reduced CTSL levels in GCD2 corneal fibroblasts. This is also supported by the finding that the exogenous CTSL expression levels reduced the mutant‐TGFBIp in GCD2 corneal fibroblasts. Although the exact mechanisms of reduced CTSK and CTSL still need to be elucidated, we here suggest that reduced TFEB in GCD2 corneal fibroblasts may lead to altered levels of CTSs, because TFEB regulates the expression of most lysosomal enzymes, including CTSs.^26^, ^27^, ^32^ Moreover, the exogenous TFEB expression reversed the levels of CTSL, including several CTSs.

Since the lysosomal alkalization is associated with chronic changes in autophagy and lysosomal degradation pathways, the elevated lysosomal pH could cause the impaired autophagic clearance of mutant‐TGFBIp and result in their accumulation in the lysosomes of GCD2 corneal fibroblasts. This interpretation is supported by two studies showing that elevated lysosomal pH caused the defective autophagic clearance of pathogenic molecules.^34^, ^35^ Collectively, these data indicate that elevated lysosomal pH may be a key factor in the accumulation of mutant‐TGFBIp in GCD2 corneal fibroblasts.

Lysosomal pH controls the maturation of lysosomal hydrolases and the processing of certain hydrolases,^36^ including CTSs.^37^ Even a minimal rise in pH is sufficient to depress lysosomal enzymatic activity and to slow down the degradation of cellular materials.^38^ These studies indicate that increased immature forms of CTSB and CTSL in GCD2 corneal fibroblasts result from elevated lysosomal pH. Moreover, while the immature forms of CTSB and CTSL were elevated in GCD2 corneal fibroblasts, the maturation of CTSD and CTSK was not different between WT and GCD2 corneal fibroblasts. These results reveal that the optimum pH of individual CTSs in activation and maturation may vary. This interpretation is also supported by previous studies that showed that the maturation of several CTSs could be affected by other CTSs^39^ and by self‐maturation.^30^

The intracellular distribution of the lysosomes found at the perinuclear area near the microtubule‐organizing centre (MTOC) and at the periphery of the cells.^31^ Lysosomes are functionally influenced by their positioning in cells.^40^ For example, perinuclear lysosomes can facilitate the fusions between lysosomes and autophagosomes, because these fusions primarily occur in the perinuclear area. Further, lysosomal pH is heterogeneous and affects lysosomal distribution.^29^ Hence, peripheral lysosomes are more alkaline than perinuclear lysosomes.^41^ Our previous study indicated that the fusions between lysosomes and autophagosomes were delayed in GCD2 corneal fibroblasts,^13^ and the data from this study show that the lysosomal pH of GCD2 corneal fibroblasts is higher than that of WT corneal fibroblasts. Accordingly, these data reveal that the increased perinuclear accumulation of lysosomes could not be related to lysosomal pH in GCD2. This interpretation is supported by a study that bafilomycin A_1_ treatments (as an elevator of lysosomal pH) did not alter lysosome positioning in GCD2 HO corneal fibroblasts (Data S5). Additionally, the exact mechanisms for the perinuclear area of the lysosomes in GCD2 corneal fibroblasts require further investigation.

We here showed a reduced level of the ATPase E subunit in GCD2 corneal fibroblasts. Although the exact mechanisms of how the V‐ATPase E subunit was reduced, and whether the reduction of this E subunit can elevate lysosomal pH remain to be elucidated, the reduced V‐ATPase E subunit may reveal evidence of an elevated pH in GCD2 corneal fibroblasts. This finding might be supported by a previous study demonstrating that the mutations of the V‐ATPase E subunit disrupt the disassembly of the V‐ATPase and affect catalytic activity.^42^ Collectively, these data indicate that elevated lysosomal pH contributes to the failure of pathogenic mutant‐TGFBIp degradation and CTS maturation and can render GCD2 corneal fibroblasts more vulnerable to toxic stimuli that cause further dysfunction.

The quantitative relations between protein and RNA are fundamental questions in molecular biology that are still not fully understood. In this study, the levels of LAMP2 showed significant changes at the protein level in GCD2 cells, but not at the mRNA level. We speculate that this difference may result from reduced protein turnover rather than from an increased rate of synthesis. Because it is possible that LAMP2 could be degraded by cathepsin L, LAMP2 levels may increase as a result of a reduction in cathepsin L in GCD2 cells. This conjecture is supported by a study that shows a reduction in LAMP2 levels in conjunction with an overexpression of cathepsin L, and an increase in LAMP2 levels in the presence of cysteine cathepsin inhibitor.^3^ In addition, we cannot rule out the possibility that increased lysosomal pH and reduced lysosomal activity affect the turnover of LAMP2.

In this study, exogenous CTSL expression reduced pathogenic mutant‐TGFBIp levels in GCD2 corneal fibroblasts but not in WT corneal fibroblasts. In contrast, CTSL expression elevated the level of LC3‐II in WT corneal fibroblasts but decreased the level in GCD2 corneal fibroblasts. Further, treatment with bafilomycin A_1_ and CTSL inhibitors suggested that CTSL level is associated with autophagic fluxes. Moreover, the levels of CTSL affected only corneal fibroblasts characterized by defective autophagy or reduced CTSL. These data indicate that CTSL can clear mutant‐TGFBIp by activating autophagy in GCD2 corneal fibroblasts. Accordingly, we here suggest that enhancing CTSL activity is a promising strategy for preventing and treating GCD2.

It is also possible that elevated LC3‐II levels in GCD2 corneal fibroblasts can be caused by reduced CTSL levels. This interpretation is supported by the finding that inhibition of CTSB or CTSL led to intracellular LC3‐II accumulation. Further, another study of LC3‐II accumulation in autophagosomes or lysosomes in the presence of CTSL inhibitor^43^, ^44^ supports this idea. Accordingly, reduced CTSL delays the degradation of mutant‐TGFBIp and LC3‐II and subsequently leads to the accumulation of LC3‐II and mutant‐TGFBIp in lysosomes and autophagolysosomes, which are the pathological features of GCD2 corneal fibroblasts. These findings support the hypothesis that the accumulation of pathogenic mutant‐TGFBIp in GCD2 corneal fibroblasts (Data S1 and another study^13^) is caused by lysosomal abnormalities and consequently could contribute to the development of cytotoxicity.

The accumulation of mutant‐TGFBIp in autophagolysosome and lysosomes was sufficient to induce toxicity in corneal fibroblasts.^13^, ^45^ Accordingly, strategies for reducing pathogenic mutant‐TGFBIp could provide therapeutic effects. This has been evaluated in a study in which *TGFBI* expression was repressed by a TGF‐β signalling inhibitor^18^ and clearance of mutant‐TGFBIp by autophagy induction in GCD2 corneal fibroblasts.^13^, ^16^, ^19^ Another study showed that lysosomes receive mutant‐TGFBIp via the endocytic pathway in corneal fibroblasts and corneal epithelial cells.^14^ However, additional studies are needed to develop this treatment strategy. Recent studies suggest a potential role for TFEB as a new therapeutic target for diverse diseases that are linked to impaired autophagy and lysosomal function. Therefore, we hypothesized that TFEB activity is involved in lysosomal abnormalities, and enhancement of TFEB can rescue the impaired autophagy‐lysosome pathway of GCD2 corneal fibroblasts. In support of this hypothesis, we found that TFEB activation and expression were reduced in GCD2 corneal fibroblasts. Furthermore, most importantly, exogenous TFEB expression not only promoted autophagosome formation (as shown by elevated LC3‐II levels) but also restored lysosomal function (as shown by up‐regulated CTSK and CTSL expression), which ultimately restored the disrupted lysosomal dysfunction.

Besides autophagy‐lysosomal systems regulation, TFEB participates in various biological functions.^46^ The present study also showed the protective effects of TFEB (such as the reduced cleavage of PARP1) against the apoptosis of GCD2 corneal fibroblasts induced by CTSL inhibitor. These findings suggest that the activation of TFEB could protect the degeneration of corneal fibroblasts of GCD2 patients. However, it is currently unknown how *TGFBI* mutation influences the level of TFEB. There is increasing evidence to support that expression of peroxisome proliferator‐activated receptor‐gamma coactivator‐1alpha (PGC‐1α) co‐activates the expression of TFEB.^47^ Recently, it was reported that PGC‐1α can robustly induce the expression of TFEB and rescue proteotoxicity in a mouse model of Huntington's disease.^48^, ^49^ These studies showed that PGC‐1α can rescue the impaired TFEB induction and significantly reduce TFEB expression by PGC‐1α reduction. Another study showed that PGC‐1α expression directly parallels the level of TFEB.^50^, ^51^ In addition, TFEB translocation from the cytosol to the nucleus was impaired in the absence of PGC‐1α.^52^ Although it remains unclear whether the pathogenic mutation causes a loss‐of‐function or gain‐of‐function of TGFBIp, Maeng et al showed that TGFBIp activates focal adhesion kinase (FAK) signalling.^53^ Furthermore, a recent study showed that phosphorylation of FAK was enhanced by TGFBIp and was suppressed significantly by an integrin α5β1 inhibitor.^54^ Considering that TGFBIp interacts with several integrins^55^ including α5β1,^56^ these data indicate that TGFBIp is an upstream regulator of the integrin/FAK/PGC‐1α signalling pathway. Accordingly, TFEB in GCD2 corneal fibroblasts can be reduced by reduced integrin/FAK/PGC‐1α signalling. Therefore, we here predict that PGC‐1α is reduced in GCD2 corneal fibroblasts and thereby reduces the level of TFEB. Collectively, TFEB is a potential therapeutic target for maintaining the physiological function of corneal fibroblasts from the dysfunction of autophagy and lysosomes. Preclinical studies are also needed to evaluate the consequence of TFEB overexpression or activation as a candidate therapeutic target for treating GCD2.

## CONFLICT OF INTEREST

Dr EK Kim is a member of the medical advisory board of the Avellino LAB in the USA. The remaining authors have no financial or proprietary interest in the materials presented herein.

## AUTHOR CONTRIBUTION

**Seung‐il Choi:** Conceptualization (equal); Data curation (equal); Formal analysis (equal); Funding acquisition (equal); Investigation (equal); Methodology (equal); Project administration (equal); Resources (equal); Software (equal); Supervision (equal); Validation (equal); Visualization (equal); Writing‐original draft (equal); Writing‐review & editing (equal). **Jong Hwan Woo:** Data curation (equal); Investigation (equal); Methodology (equal); Project administration (equal); Software (equal); Visualization (equal). **Eung Kweon Kim:** Conceptualization (equal); Data curation (equal); Formal analysis (equal); Funding acquisition (equal); Investigation (equal); Methodology (equal); Project administration (equal); Resources (equal); Software (equal); Supervision (equal); Validation (equal); Visualization (equal); Writing‐original draft (equal); Writing‐review & editing (equal).

## Supporting information

Supplementary Material

## Data Availability

I confirm that I have included a citation for available data in my references section, unless my article type is exempt.
