# Supplementary material for: Lysosomal dysfunction of corneal fibroblasts underlies the pathogenesis of Granular Corneal Dystrophy Type 2 and can be rescued by TFEB
Source: J Cell Mol Med. 2020 Jul 15;24(18):10343–55. doi: 10.1111/jcmm.15646 (PMC7521267; doi:10.1111/jcmm.15646)
Supplement: Supplementary file 1 — Supplementary Material [file JCMM-24-10343-s001.docx]

**SUPPORTING INFORMATION**

1. **SUPPORTING TABLES**

**Supporting table 1. Antibodies used in this study**

| **Antibodies** | **Provider** | **Catalog Number** | **Experiment** | **Results**  **(Figure)** |
| --- | --- | --- | --- | --- |
| Anti-Cathepsin B | Calbiochem, San Diego, CA, USA. | PC41 | WB* | 1, 7 |
| Anti-Cathepsin D | Calbiochem, San Diego, CA, USA. | IM16 | WB | 1, 7 |
| Anti-Cathepsin K | Abcam, Cambridge, UK. | ab19027 | WB | 1 |
| Anti-Cathepsin L | Abcam, Cambridge, UK. | ab6314 | WB | 1 |
| Anti-GAPDH | Merck Millipore, Billerica, MA, USA. | MAB374 | WB | 1, 2, 4, sup-4, sup-3 |
| Anti-Cathepsin D | Calbiochem, San Diego, CA, USA. | IM16 | IFCM ** | 1 |
| Anti-Cathepsin K | SantaCruz Biotechnology Inc., Santa Cruz, CA, USA. | sc-48353 | IFCM | 1 |
| Anti-Cathepsin L | R&D Systems, Minneapolis, MN, USA. | AF952 | IFCM | 1 |
| Anti-V-ATPase E | SantaCruz Biotechnology Inc., Santa Cruz, CA, USA. | sc-20946 | WB | 2, 7 |
| Anti-LAMP2 | Development Studies Hybridoma Bank, Iowa City, IA, USA. | H4B4 | WB, IFCM | 3, 7 |
| Anti-TGFBIp | R&D Systems, Minneapolis, MN, USA. | AF2935 | WB | 4, 5, sup-2 |
| Anti-LC3B | Cell Signaling Technology  Beverly, MA, USA. | 3868S | WB | 5, 7, sup-1, sup-3 |
| Anti-β-actin | Sigma‐Aldrich, St. Louis, MO, USA. | A5441 | WB | 3, 5 |
| Anti-p62 | Cell Signaling Technology  Beverly, MA, USA. | 5114S | WB | sup-3 |
| Anti-Cathepsin L | SantaCruz Biotechnology Inc., Santa Cruz, CA, USA. | Sc-32801 | WB | 5, 7 |
| Anti-PARP1 | Cell Signaling Technology  Beverly, MA, USA. | 9532S | WB | 7, sup-5 |
| Anti-Caspase 3 | Cell Signaling Technology  Beverly, MA, USA. | 9662 | WB | 7 |
| Anti-Cleaved Caspase 3 | Cell Signaling Technology  Beverly, MA, USA. | 9661 | WB | 7 |
| Anti-TFEB | Bethyl Laboratories Inc, Montgomery, TX, USA. | A303-673A | WB | 6, 7 |
| Anti-14-3-3 | SantaCruz Biotechnology Inc., Santa Cruz, CA, USA | sc-628 | WB | 6 |
| Anti-Histone H3 | Calbiochem, San Diego, CA, USA. | ab1791 | WB | 6 |
| Anti-TFEB | R&D Systems, Minneapolis, MN, USA. | MAB9170 | IFCM | 6, 7 |
| Anti-cathepsin K | SantaCruz Biotechnology Inc., Santa Cruz, CA, USA. | sc-48353 | WB | 7 |
| Goat anti-Rabbit IgG (H+L) Secondary Antibody, HRP | Thermo Fisher Scientific Inc., Waltham, MA, USA. | 31460 | WB |  |
| Goat anti-Mouse IgG (H+L) Secondary Antibody, HRP | Thermo Fisher Scientific Inc., Waltham, MA, USA. | 31430 | WB |  |
| Goat anti-Mouse IgG (H+L) Cross-Adsorbed Secondary Antibody, Alexa Fluor 488 | Thermo Fisher Scientific Inc., Waltham, MA, USA. | A-11001 | IFCM |  |
| Goat anti-Rabbit IgG (H+L) Highly Cross-Adsorbed Secondary Antibody, Alexa Fluor 488 | Thermo Fisher Scientific Inc., Waltham, MA, USA. | A-11034 | IFCM |  |
| Donkey anti-Goat IgG (H+L) Cross-Adsorbed Secondary Antibody, Alexa Fluor 488 | Thermo Fisher Scientific Inc., Waltham, MA, USA. | A-11055 | IFCM |  |
| Peroxidase-AffiniPure Rabbit Anti-Goat IgG (H+L) | Jackson ImmunoResearch Laboratories, Inc., West Grove, PA, USA. | 305‐035‐003 | WB |  |
| Goat anti-Mouse IgG (H+L) Cross-Adsorbed Secondary Antibody, Alexa Fluor 546 | Thermo Fisher Scientific Inc., Waltham, MA, USA. | A-11003 | IFCM |  |

WB*: Western blots, IFCM**: Immunofluorescence confocal microscopy

Sup: Supplementary data

**Supporting table 2. Materials used in this study**

| **Name** | **Provider** | **Catalog Number** | **Concentration**  **Used** | **Results**  **(Figure)** |
| --- | --- | --- | --- | --- |
| Acridine Orange Hydrochloride | Sigma‐Aldrich, St. Louis, MO, USA. | A8097 | 20 μg/ml | 2 |
| LysoSensor™ Yellow/Blue DND-160 | Thermo Fisher Scientific Inc., Waltham, MA, USA. | L7545 | 1 μM | 2 |
| Cathepsin L inhibitor | SantaCruz Biotechnology Inc., Santa Cruz, CA, USA. | sc-3132 | 50-250 μM | 4 |
| CA-074 methyl ester (CTS B inhibitor) | SantaCruz Biotechnology Inc., Santa Cruz, CA, USA. | sc-214647 | 100 μM | 4 |
| Pepstatin A (CTS D inhibitor) | SantaCruz Biotechnology Inc., Santa Cruz, CA, USA. | sc-45036 | 100 μM | 4 |
| BML-244 (CTS K inhibitor) | SantaCruz Biotechnology Inc., Santa Cruz, CA, USA. | sc-221370 | 100 μM | 4 |
| Cathepsin L from human liver (enzyme) | Sigma‐Aldrich, St. Louis, MO, USA. | C6854 | 0.25-2.5 mU | 4 |
| Recombinant human LC3B protein | Abcam, Cambridge, UK | ab103506 | 1 μg | sup-3 |
| Lentivirus-Cathepsin L | Watson R&D shearing, South Korea. |  |  | 5 |
| Bafilomycin A1 | Sigma‐Aldrich, St. Louis, MO, USA. | B1793 | 0.01~0.03 μM | 5, sup-4 |
| Presto Blue | Invitrogen, Life Technologies, USA. | A13261 |  | sup-5 |
| NE-PER™ Nuclear and Cytoplasmic Extraction Reagents | Thermo Fisher Scientific Inc., USA. | 78835 |  | 6 |
| Dynabeads™ M-280 Sheep Anti-Rabbit IgG | Thermo Fisher Scientific Inc., USA. | 11204D |  | 6 |
| Lentivirus-TFEB | Watson R&D shearing, South Korea. |  |  | 7 |

Suppl: Supplementary data

**Supporting table 3. Primers for q-PCR**

| **Gene Name** | **Forward Primer** | **Reverse Primer** | **Product Size (bp)** | **Results**  **(Figure)** |
| --- | --- | --- | --- | --- |
| CTSB | 5`-AGAATGGCACACCCTACTGG-3` | 5`-TGCATTTCTACCCCGATCTC-3` | 212 | 1 |
| CTSD | 5`-GACACAGGCACTTCCCTCAT-3` | 5`-CTCTGGGGACAGCTTGTAGC-3` | 174 | 1 |
| CTSK | 5`-TTCTGCTGCTACCTGTGGTG-3` | 5`-CCAGGTGGTTCATAGCCAGT-3` | 219 | 1 |
| CTSL | 5`-GTCTACCCCGAACTCTGCTG-3` | 5`-CCTTCCACTTGGTCCACTGT-3` | 228 | 1 |
| *LAMP2* | 5`-GGTTAATGGCTCCGTTTTCA-3` | 5`-TCATCCAGCGAACACTCTTG-3` | 201 | 3 |
| ACTB | 5`-GGCATCCTCACCCTGAAGTA-3` | 5`-AGGTGTGGTGCCAGATTTTC-3` | 82 | 1 |

1. **SUPPORTING METHODS**

**Supporting method 1. CTSL and TFEB retrovirus plasmid construction and transduction**

Each of CTSL and TFEB genes was subcloned at the EcoRI-XhoI sites of the pLVX-IRES-tdTomato Vector (CLONTECH Laboratories Inc., Palo Alto, California, USA) using standard recombinant DNA technology. Retrovirus particles were generated by WatsonRnd Sharing Co. Ltd. (South Korea). Briefly, HEK-293T cells were cultured and transfected with pLVX-IRES-tdTomato-TFEB or pLVX-IRES-tdTomato CTSL, using Lipofectamine 2000, according to the manufacturer’s instructions (Invitrogen). After 12 h, the medium was replaced with a new medium and incubated for 48 h. Retrovirus particles were collected by removing the medium and filtering it with a 0.45 µm syringe filter. The corneal fibroblasts were infected with Lenti-CTSL and Lenti-TFEB at a confluence of 30–50 %. The medium was then changed, the corneal fibroblasts incubated for a further 2 days, and passaged for further experiments.

**Supporting method 2. Preparation of cell lysates, Western blots, and immunoprecipitation analysis**

Cell lysates were centrifuged at 10 000 x *g* for 10 minutes at 4 °C. The supernatant was used to determine the total protein concentration with a bicinchoninic acid (BCA) kit (Pierce). Total cellular proteins (~50 µg) were electrophoresed in NuPAGE™ 4-12 % Bis-Tris Protein Gels (Invitrogen: NP0335), at 70~120 V in NuPAGE® MES SDS running buffer (Life Technologies: NP0002). Proteins were transferred to polyvinylidene difluoride membranes (PVDF) with the iBlot 2 Dry Blotting System (Thermo Fisher Scientific), blocked in 5 % dry milk in TBS-T (0.02 mol/L Tris/ 0.15 mol/L NaCl, pH 7.5, containing 0.1 % Tween 20) at RT for 1 hour, washed three times with TBS-T, and then incubated with primary antibodies (supplementary table 1). After washing three times with TBS-T, the blots were incubated with secondary antibodies conjugated with horseradish peroxidase at RT for 1 hour. Horseradish peroxidase-linked anti-mouse IgG or anti-rabbit IgG was used as a secondary antibody (supplementary table 1). Immunoblots were visualized using the SuperSignal West Pico Chemiluminescent Substrate that was obtained from Pierce (supplementary table 2). The intensities of the immunoreactive bands were quantified using computer software (ImageJ software, version 1.37, Wayne Rasband, NIH), corrected by background subtraction, and normalized to the intensity of the corresponding β-actin or GAPDH (glyceraldehyde-3-phosphate dehydrogenase) protein bands.

**Supporting method 3. Immunofluorescence staining and confocal microscopy**

Cells were washed with phosphate-buffered saline (PBS) and blocked with 1 % BSA (Sigma-Aldrich, A3311) in PBS for 60 min, then incubated with the primary antibody in 1 % BSA in TBS-T for 1 h, at room temperature (RT) or overnight at 4 °C. Cells were hybridized with secondary antibodies for 1 h at RT. The coverslips were mounted on glass slides using Vectashield mounting medium (Vector Labs Inc., H-1200). Cells were viewed under the Zeiss LSM 700 confocal microscope (Carl Zeiss, Jena, Germany).

**Supporting method 4. Subcellular fractions**

Briefly, corneal fibroblasts were harvested by centrifugation for 5 min at 500 $\times$ g and washed by suspending the cell pellet with PBS. After CER I buffers were added to the cell pellet, it was centrifuged, and the supernatants (cytoplasmic extract) were transferred to a pre-chilled tube. The pellet, containing the nuclei, was suspended in the ice-cold CER II buffer. The pellet was centrifuged, and then the supernatants (nuclear extract) were transferred to a pre-chilled tube. Protein concentrations of the two fractions were assayed using the BCA protein assay kit (Thermo Fisher Scientific Inc., Waltham, MA, USA).

## Supporting method 5. In vitro cleavage assays of TGFBIp and LC3

## To generate TGFBIp and the mutant-TGFBIp product, WT and GCD2 HO corneal fibroblasts were chased in serum-free DMEM at 37°C for 2 days, and the media containing TGFBIp were collected for in vitro digestion assays. Collected medium containing TGFBIp or recombinant LC3 protein was incubated with CTSL (0.25–2.5 units) for 2 h at 37°C in reaction buffer (400 mM sodium acetate, pH 5.5, with 4 mM EDTA and 8 mM DTT). After incubation, digestion reactions were immediately stopped by adding SDS sample buffer, and the samples were loaded onto 4–12% NuPAGE Bis-Tris precast gels and the bands were visualized by western blotting.

**Supporting method 6. Cell viability**

Corneal fibroblasts were plated in 96-well plates at a density of 10 000 cells/well and incubated overnight. After 16 h incubations, with various concentrations of CTS inhibitors, the PrestoBlue Cell Viability Assay reagent (Invitrogen) was used to determine the number of viable cells. Briefly, the culture medium was removed, and 10 μl PrestoBlue reagents were added to each well in the 90 μl culture medium. Cultures were incubated at 37 °C for 2~4 h under 95 % humidity and 5 % CO_2_. Optical density was measured at 490 nm using a microplate reader (VERSAmax, Molecular Devices). The PrestoBlue reagent is reduced by metabolically active cells to a red color that is highly fluorescent. The fluorescence (excitation 560 nm, emission 590 nm) was measured after 2~4 h incubation with the PrestoBlue reagent.

1. **SUPPORTING DATA**

**Supporting data 1**


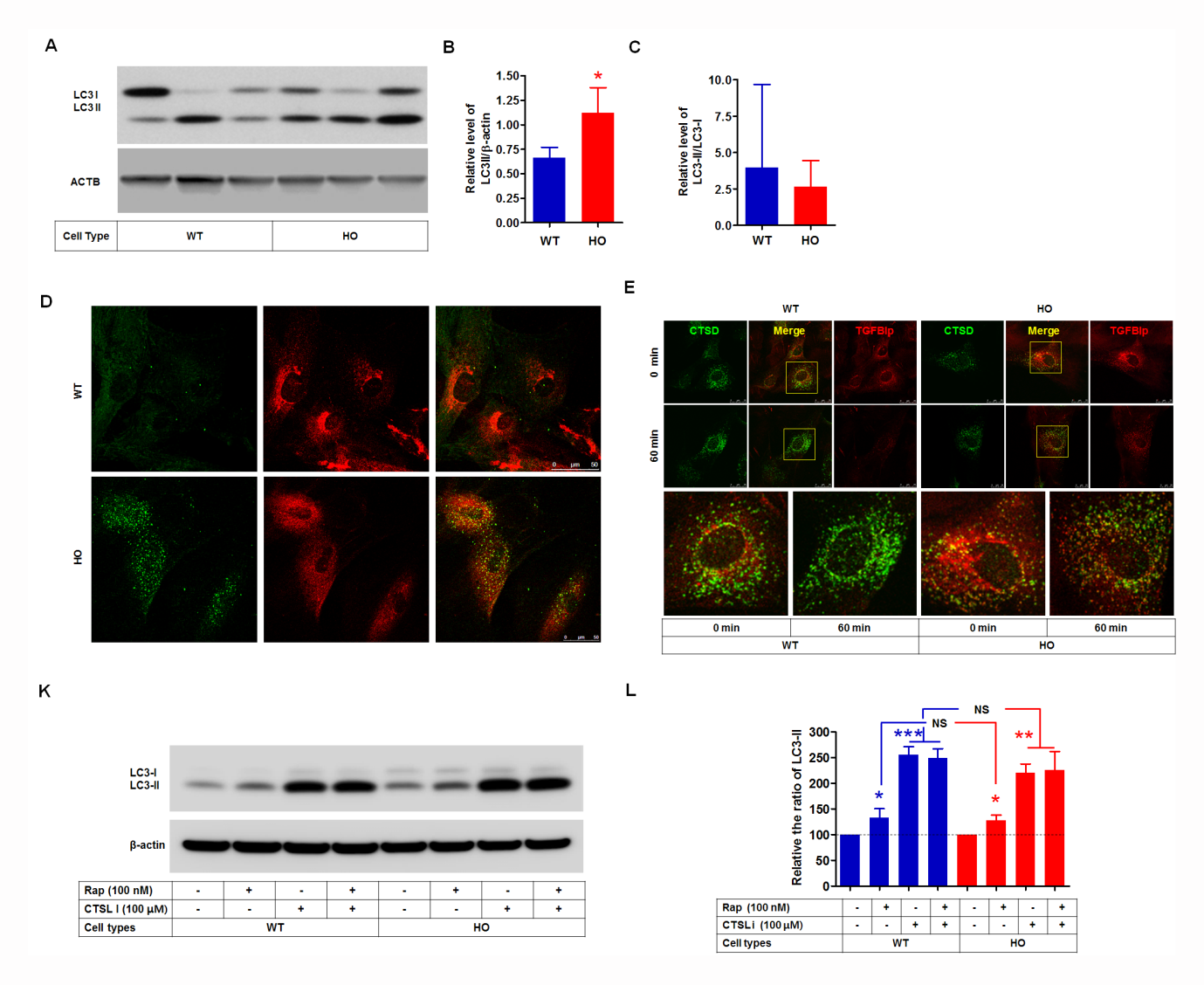


**Sup. data 1.** The delayed degradation of the mutant-TGFBIp within the lysosomes and the autophagolysosome. **(A)** Western blots analysis of LC3-II in WT and HO corneal fibroblasts. **(B)** Quantification of the relative band intensities of the LC3-II in (**A**). Data were normalized against β-actin. **(C)** Quantification of the relative band intensities of the LC3-II in (**A**). Data were normalized against LC3-I. Differences between the values were analyzed by the Student t-test with **P* ≤ 0.05. **(D)** Representative images of the LC3 (autophagosome marker) staining in the WT and GCD2 corneal fibroblasts. **(E)** WT and GCD2 HO corneal fibroblasts were incubated with 100 μg/ml CH X for 60 min and then fixed and double stained with anti-TGFBIp and anti-CTSD. Images were obtained with a confocal microscope separately for TGFBIp and CTSD. WT-TGFBIp completely disappeared after 60 min, and it could not be observed inside the cell. In contrast, mutant-TGFBIp was colocalized with lysosomes and was maintained even after 60 min. **(F)** Western blots analysis of LC3-II in WT and HO cells that were treated with inhibitors of CTSL in presence (+) or absences (-). CTSL i: CTSL inhibitor. **(G)** Quantification of the relative band intensities of the LC3-II in **(F)**. Data were normalized against β-actin. Differences between the values were analyzed by the Student t-test with **P* ≤ 0.05, ***P* ≤ 0.01, ****P* ≤ 0.001.

**Supporting data 2**


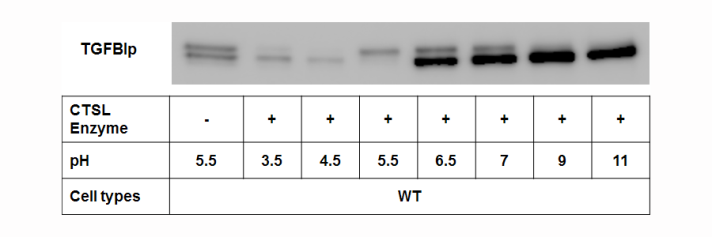


**Sup. data 2.** Western blots of TGFBIp incubated with CTSL enzyme *in vitro*. TGFBIp were incubated with 2.5 m units of CTSL enzyme for 2 h at 37 °C in pH-dependent manner (pH 3.5, 4.5, 5.5, 6.5, 7.0, 9.0, and 11, respectively). -: untreated; +: treated; CTSL i: CTSL inhibitor; m unit: mile unit.

**Supporting data 3**


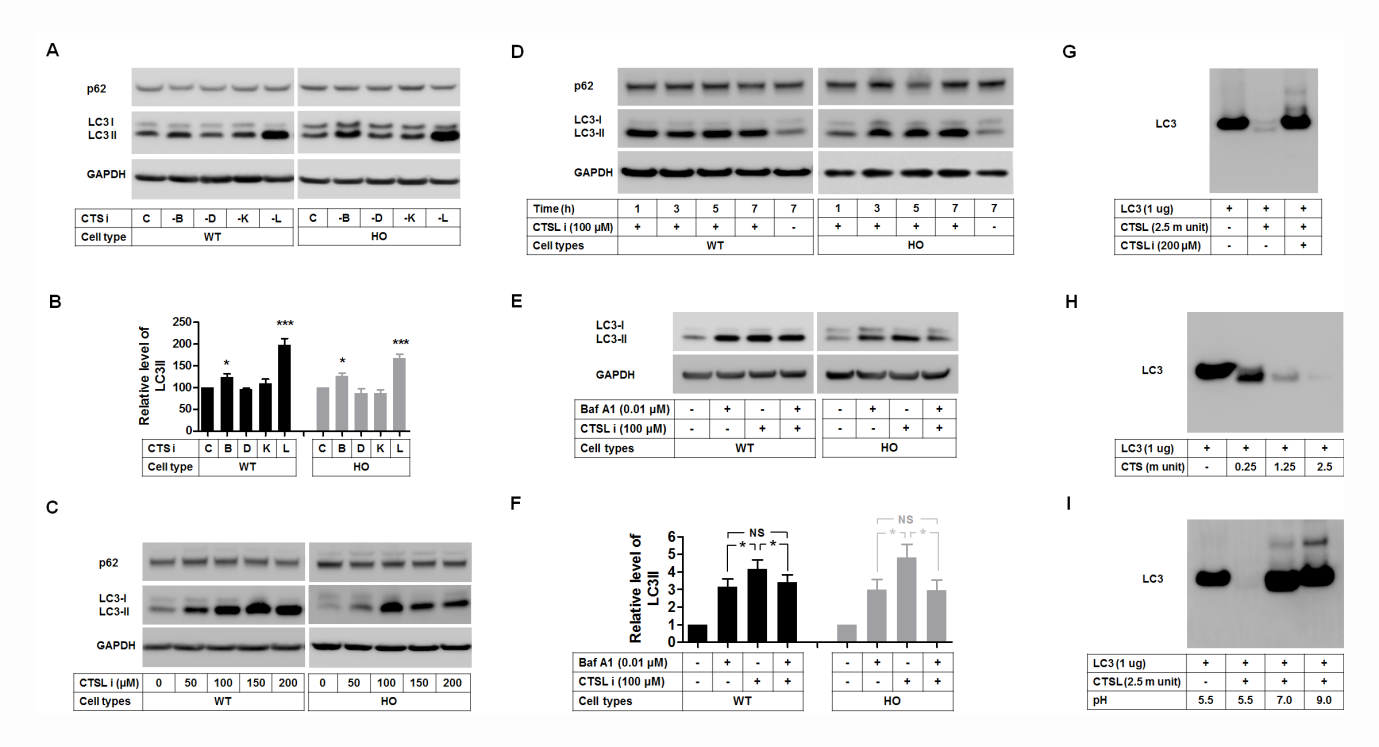


**Sup. data 3**. CTSs degrade autophagosomal LC3-II. **(A)** Western blots analysis of LC3-II and SQSTM1/p62 in the WT and HO cells that were treated with inhibitors of the CTSB, CTSD, CTSK, and CTSL. CTS i: CTS inhibitor. **(B)** Quantification of the relative band intensities of the LC3-II in **(A)**. Differences between the values were analyzed by the Student t-test with **P* ≤ 0.05 and ****P* ≤ 0.001. **(C)** A dose-dependent increase of the LC3-II by CTSL i in the WT and GCD2 HO corneal fibroblasts. Western blots analysis of the LC3-II in the WT and GCD2 HO cells, treated with four different concentrations of CTSL i (50, 100, 150, and 200 μM). **(D)** Time-dependent effect of CTSL i on the levels of the LC3-II in the WT and GCD2 HO corneal fibroblasts. CTSL i: CTSL inhibitor; h: hour. **(E)** The effects of the Baf A_1_ and CTSL i on the level of LC3-II in the WT and GCD2 HO corneal fibroblasts. LC3-II was analyzed with Western blots for the total protein extracted from the WT and HO cells that are treated with CTSL i and/or Baf A_1_ for 16 h. CTSL i: CTSL inhibitor; Baf A_1_: Bafilomycin A_1_ **(F)** Quantification of the relative band intensities of the LC3-II in **(E)**. Differences between the values were analyzed by the Student t-test with **P* ≤ 0.05. NS: not significant. **(G~I)** Western blots of LC3 incubated with CTSL proteins in vitro. LC3 were incubated with 2.5 m units of CTSL for 2 h at 37 °C in pH 5.5, with or without CTSL i **(G)**, CTSL dose-dependent manner **(H)** and pH-dependent manner **(I)**. -: untreated; +: treated; CTSL i: CTSL inhibitor; m unit: mile unit.

**Supporting data 4**

**
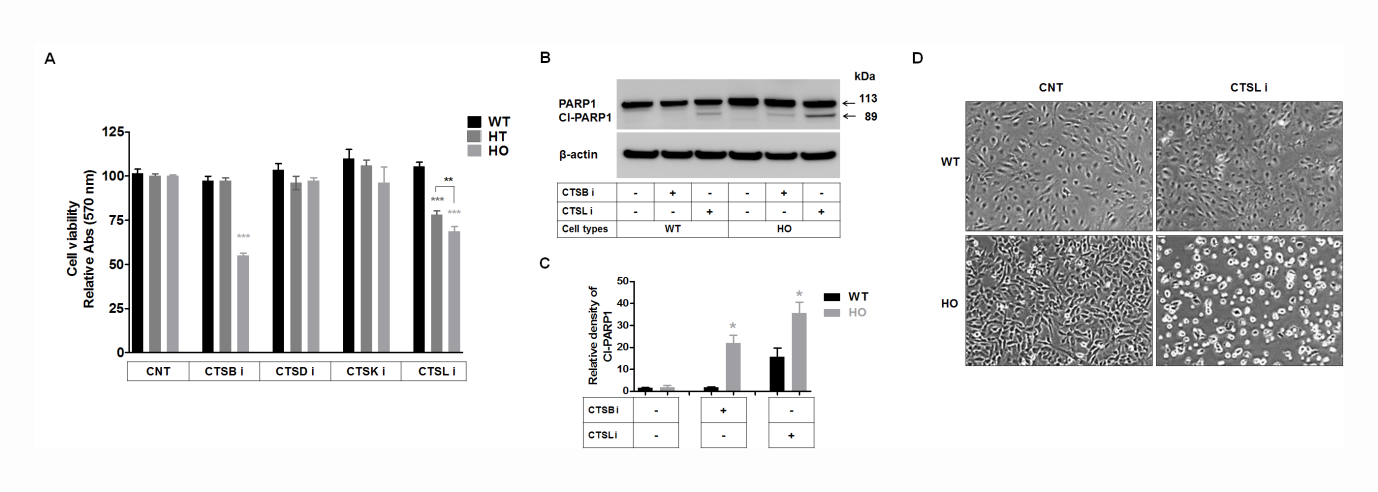
Sup. data 4.** Death of the GCD2 corneal fibroblasts is induced by the inhibition of the CTSB and CTSL. **(A)** Cell viability analysis in the WT and GCD2 corneal fibroblasts treated with inhibitors of the CTSL and CTSB. WT, HT, and HO corneal fibroblasts were exposed to CTSs i for 16 h, and the quantitative cell viabilities were estimated using PrestoBlue Cell Viability Reagent. Error bars: SD from three independent experiments. **P* ≤ 0.05. **(B)** Apoptosis analysis in the WT and GCD2 HO corneal fibroblasts treated with inhibitors of CTSL and CTSB for 16 h. PARP1 and ACTB were determined by Western blots. Cleaved PARP1 had two major fragments, migrating at 113 kDa and 89 kDa. **(C)** Quantification of the relative band intensities of Cl-PARP1 in **(B)**. Different values between the WT and HO corneal fibroblasts were analyzed by the Student t-test with **P* ≤ 0.05. Cl-PARP1: Cleaved PARP1. **(D)** Representative phase-contrast photomicrographs of the CTSL i-treated WT and HO corneal fibroblasts. X 200.

**Supporting data 5**

**
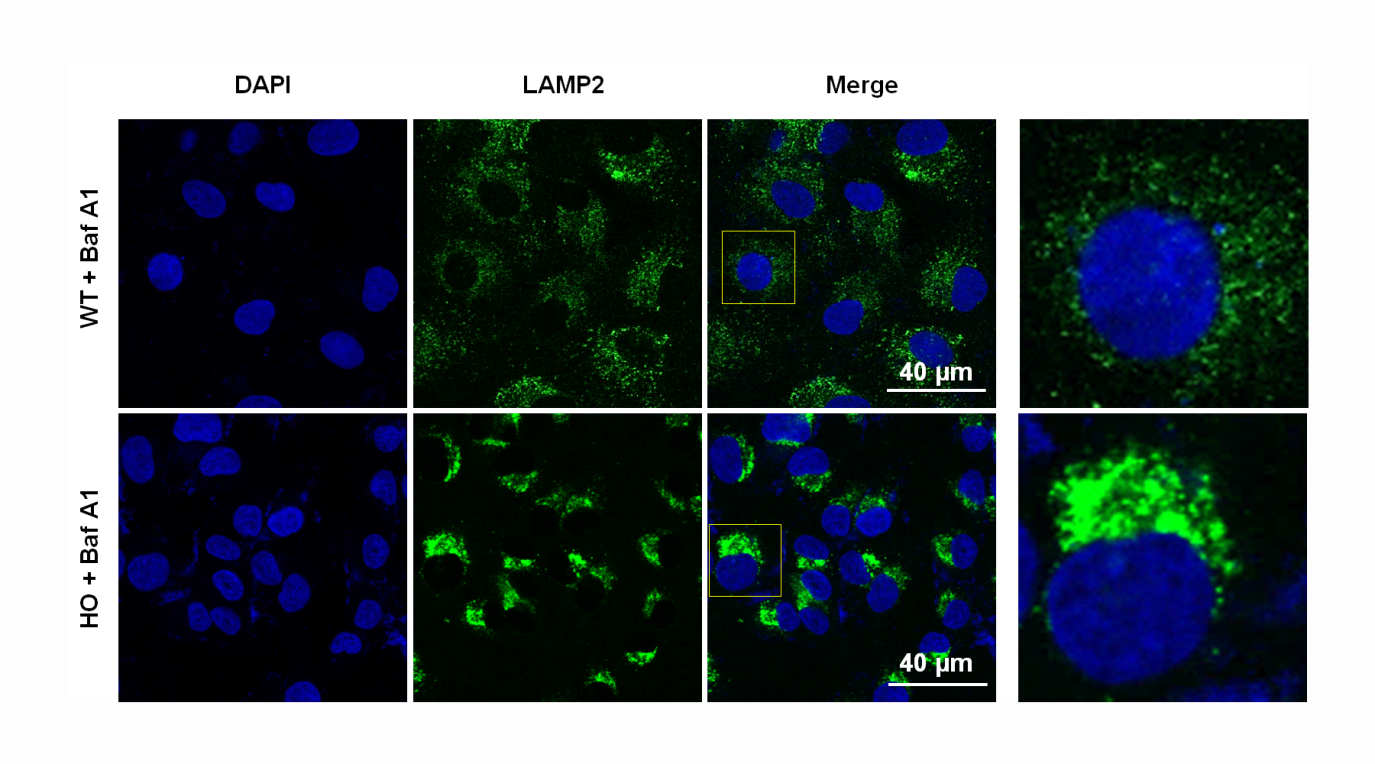
**

**Sup. data 5.** The analysis of lysosomal distributions in the GCD2 corneal fibroblasts with the bafilomycin A_1_ treatments. Representative images of the LAMP2 (lysosomal marker) staining in the WT and GCD2 cells under bafilomycin A_1_ treatments. Cells were methanol-fixed and immunostained with anti-LAMP2 (green) and counterstained with DAPI (blue: nuclear marker).
